# Negative Correlation between the Diffusion Coefficient and Transcriptional Activity of the Glucocorticoid Receptor

**DOI:** 10.3390/ijms18091855

**Published:** 2017-08-25

**Authors:** Shintaro Mikuni, Johtaro Yamamoto, Takashi Horio, Masataka Kinjo

**Affiliations:** Laboratory of Molecular Cell Dynamics, Faculty of Advanced Life Science, Hokkaido University, Sapporo 0010021, Japan; shintaro.mikuni@nitto.com (S.M.); jyamamoto@eis.hokudai.ac.jp (J.Y.); serenade.k.v.525@gmail.com (T.H.)

**Keywords:** raster image correlation spectroscopy, green fluorescence protein, nuclear receptor, diffusion analysis, transcription

## Abstract

The glucocorticoid receptor (GR) is a transcription factor, which interacts with DNA and other cofactors to regulate gene transcription. Binding to other partners in the cell nucleus alters the diffusion properties of GR. Raster image correlation spectroscopy (RICS) was applied to quantitatively characterize the diffusion properties of EGFP labeled human GR (EGFP-hGR) and its mutants in the cell nucleus. RICS is an image correlation technique that evaluates the spatial distribution of the diffusion coefficient as a diffusion map. Interestingly, we observed that the averaged diffusion coefficient of EGFP-hGR strongly and negatively correlated with its transcriptional activities in comparison to that of EGFP-hGR wild type and mutants with various transcriptional activities. This result suggests that the decreasing of the diffusion coefficient of hGR was reflected in the high-affinity binding to DNA. Moreover, the hyper-phosphorylation of hGR can enhance the transcriptional activity by reduction of the interaction between the hGR and the nuclear corepressors.

## 1. Introduction

The glucocorticoid receptor (GR) is a transcription factor that controls broad physiological gene networks and has pathological effects in a range of diseases; therefore, this protein offers an excellent target for therapeutic intervention [1,2,3,4]. The functional domains of GR consist of an N-terminal transactivation domain, a central DNA binding domain, and a C-terminal ligand binding domain [1,5]. The N-terminal domain has a strong transcriptional activation function (AF-1), which allows for the recruitment of coregulators. The second activation function domain (AF-2), which interacts with coregulators, is located on the C-terminal ligand binding domain. In the absence of ligands, GR forms a transcriptionally inactivated complex with chaperone and cochaperone proteins, such as Hsp90, Hsp70, p23, and Hop in the cytoplasm [6]. Upon ligand binding, GR is driven into the nucleus, where it regulates transcription of target genes. The processes of transcriptional regulation mediated by GR in the nucleus have been investigated [1,7]. In one process, GR mainly exerts its transcriptional activation or repression by direct high-affinity binding to GR binding regions (GBR), including the palindromic glucocorticoid response elements (GRE) or negative GRE (nGRE) of target genes. In another, GR indirectly represses the gene transcription via tethering to other transcription factors, such as activator protein 1 (AP-1) and nuclear factor κB (NF-κB), which regulates pro-inflammatory transcription. GR-mediated transcriptional regulation is controlled not only by ligand binding but also by post-translational modifications, which include phosphorylation, acetylation, ubiquitination, and SUMOylation [8]. Phosphorylation of GR is a key process in the regulation of GR function, such as transcriptional activity, nuclear-cytoplasmic shuttling, and interaction with cofactors and other transcriptional factors [9,10]. In human GR (hGR), 11 phosphorylated amino acids (serines and tyrosines) were identified; interestingly, eight of them are located in the N-terminal AF-1 of hGR [10].

By binding to other partners, such as DNA and transcription factors, GR alters its diffusion properties in the cell nucleus. For this reason, the mobility of GFP fused GR has been analyzed using fluorescence recovery after photobleaching (FRAP) [11,12,13,14,15,16,17], fluorescence correlation spectroscopy (FCS) [17,18,19,20], and single-molecule microscopy [13] to investigate GR function in live cells. Many FRAP studies have clarified that the mobility of GR is correlated with its transcriptional activities [14,15,16]. While FRAP is a useful technique to determine the mobile and immobile fractions, it requires careful determination of the beam profile to calculate the diffusion coefficients and careful modeling and fitting to extract multiple diffusion components. Alternatively, FCS can be used to quantify the diffusion coefficient of both single and multiple components [21]. However, classical FCS as well as FRAP provides information in a single-spot, which is not sufficient to analyze the diffusion property in a spatially heterogeneous distribution, such as GR in nucleus [22].

**Figure 1:**
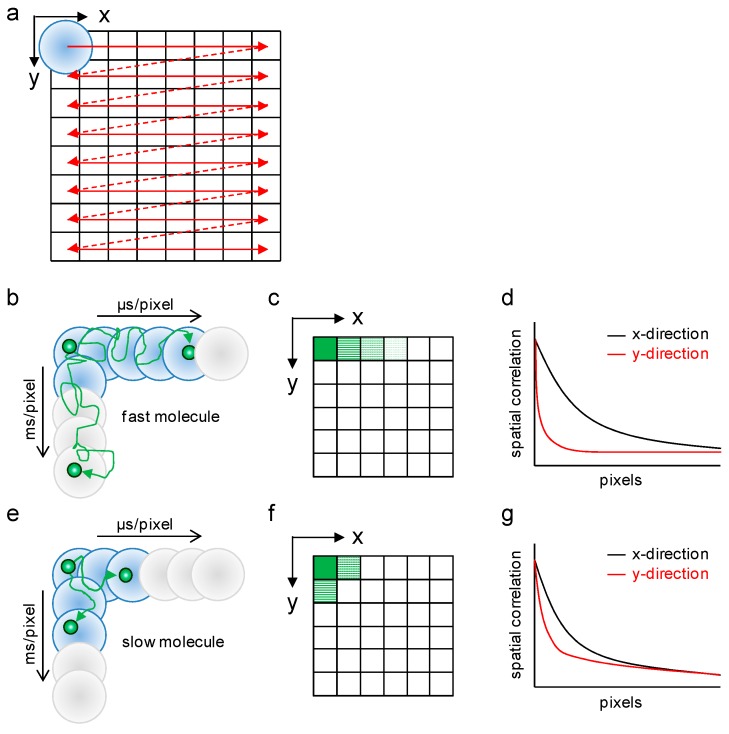
The principle of laser scanning microscopy (LSM) imaging for raster image correlation spectroscopy (RICS) analysis. (**a**) LSM images consist of pixels from line-by-line scanning. The detectable fluorescent signal (blue circles) and undetectable cases (gray circle) of: (**b**) fast; and (**e**) slow moving molecules (green circles) from scans result in the LSM image ((**c**,**f**), respectively). The spatial correlation of: (**d**) fast; and (**g**) slow moving molecules. Black and red lines represent the correlations calculated from the *x*- and *y*-direction pixels, respectively.

In this study, we quantified the diffusion property of hGR in live cell nuclei by raster image correlation spectroscopy (RICS). RICS is a noninvasive image analysis technique [23,24] to analyze the diffusion coefficient and number of fluorescently labeled biomolecules in live cells from laser scanning microscopy (LSM) images. The LSM image was taken with line-by-line scanning of a laser spot, which was generated by a confocal optics system (Figure 1a). Therefore, the pixels in LSM images not only have spatial information represented by the fluorescent intensity at the position but also have temporal information from the time lag according to raster scanning speed. In general conditions, the time lag of each pixel is a microsecond in the horizontal scanning direction (*x*-direction) and a millisecond in the vertical scanning direction (*y*-direction). In the case of observation for fast moving molecules that had a high diffusion coefficient, the fluorescent signal could be detected along some pixels in the *x*-direction, but less in the *y*-direction (Figure 1b,c) because scanning speed in the *y*-direction is too slow to chase the fast moving molecule. Consequently, the spatial correlation represents gentle-decay in the *x*-direction but a steep-decay in *y*-direction (Figure 1d). Conversely, slow moving molecules that had a low diffusion coefficient gave less pixels in *x*-direction but more in *y*-direction (Figure 1e,f). As a result, the decay of the spatial correlation becomes steeper in *x*-direction and gentler in *y*-direction (Figure 1g). Finally, the diffusion coefficient and number of fluorescent molecules can be obtained by global fitting on both the *x*- and *y*-directional spatial correlations in the same manner as that by fluorescence correlation spectroscopy (FCS) and the other fluorescence fluctuation analysis techniques.

RICS has been applied to analyze membrane proteins [25,26], DNA [27], and other biomolecules [28,29] because it provides the spatial distribution of the diffusional coefficients in the diffusion map [27,30]. Each brick constituting the diffusion map showed the diffusion property of biomolecules contained in each microdomain. This mapping analysis in RICS, in other words, has a multipoint diffusion analysis in one of the most useful and easiest methods for quantifying the heterogeneous distribution of biomolecules in live cells.

Here, we analyzed the diffusion property of EGFP labeled hGR (EGFP-hGR) wild type and the various transcriptional mutants by RICS and showed that the diffusion coefficient of hGR negatively and strongly correlated with its transcriptional activities. Furthermore, hyper-phosphorylation can change the diffusion property and enhance the transcriptional activity of hGR.

## 2. Results

### 2.1. Analysis of Diffusion Properties of the Glucocorticoid Receptor by Raster Image Correlation Spectroscopy (RICS)

**Figure 2:**
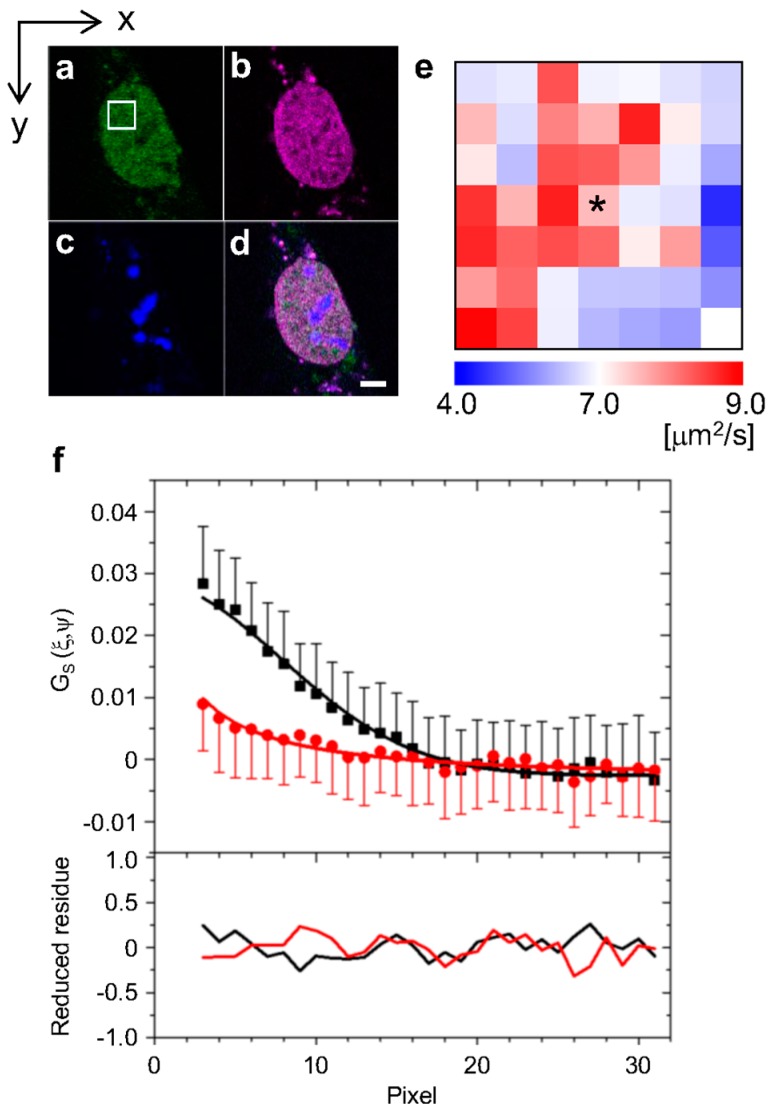
LSM image for RICS analysis. The LSM images are: (**a**) EGFP-hGR^WT^; (**b**) H2B-mCherry; (**c**) TagBFP-fibrillarin; and (**d**) the merged image. The scale bar is 5 μm. The white square in (**a**) indicates the region for RICS analysis, the size of which was 5.38 × 5.38 μm. (**e**) The diffusion map with 7 × 7 bricks in the region represented the white square in (**a**) by RICS analysis. (**f**) The spatial correlation function and fitting (upper) and the reduced residue (lower) of the brick indicated with an asterisk (*) in (**e**). The black and red points indicate the spatial correlation function of *x*- and *y*-direction of images, respectively. Black and red lines indicate the fit by Equation (1).

To analyze the diffusion properties of the human glucocorticoid receptor (hGR) in the cell nucleus after stimulation with the agonist Dexamethasone (Dex), raster image correlation spectroscopy (RICS) was carried out. The region for RICS observations was determined in a nucleus, excluding the nucleolus, by the confocal LSM image of a cell expressing EGFP-hGR wild type (EGFP-hGR^WT^, Figure 2a, white square). The localization in the nucleus and nucleolus were visualized by expressing the mCherry-fused histone 2B (H2B-mCherry) and TagBFP-fused fibrillarin (TagBFP-fibrillarin) protein, respectively (Figure 2b,c). Figure 2d is the merged image. Followed by RICS analysis of the one hundred frames shown as white squares in Figure 2a, the 7 × 7 bricks diffusion map as shown in Figure 2e was constructed. In this map, each brick represented the microdomain in the nucleus, which corresponded to a 0.7 × 0.7 μm area. In the presence of Dex, the EGFP-hGR^WT^ was distributed with heterogeneous diffusion coefficients (Figure 2e) and numbers (Figure S1a) in the nucleus. The typical spatial correlation function, which was obtained from the brick is indicated with an asterisk (*) in the diffusion map (Figure 2e), is shown in Figure 2f. The spatial correlation function was successfully fit with a one-component diffusion model to calculate the diffusion coefficient because the reduced χ^2^ values of all bricks were within 1.000 ± 0.025 as shown in Figure S1b.

**Figure 3:**
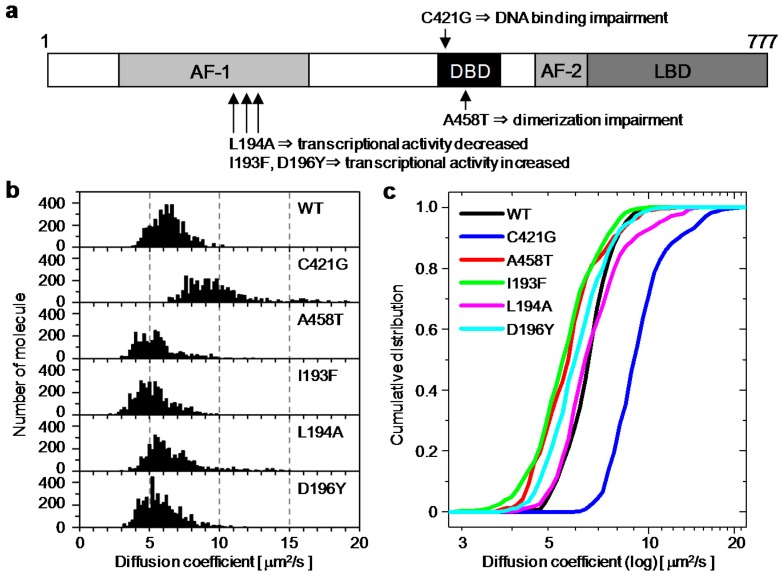
The histograms and cumulative distributions of diffusion coefficients for EGFP-hGR wild type and mutants: (**a**) The functional domain of the glucocorticoid receptor and the position of the mutations; (**b**) the diffusion coefficient histograms of hGR wild type and mutants were constructed by integrating the numbers which were calculated by one-component fitting analysis of each brick in the diffusion map (*n* = 6–8 cells); (**c**) the cumulative distributions constructed from the diffusion coefficient histograms of hGR wild type and mutants.

To analyze the distribution of the diffusion coefficients, the diffusion properties among hGR wild type and five mutants (Figure 3a) were used: C421G and A458T are point mutation that impair DNA binding and dimerization [8], respectively; I193F and D196Y mutations increased the transcriptional activities of hGR; and L194A decreased transcriptional activities of hGR [31]. The histograms of the diffusion coefficients were constructed by integrating the number of molecules in each brick of the diffusion map, which was obtained from 6–8 cells expressing EGFP-hGR wild type or mutants (Figure 3b). As shown in Figure 3c, the cumulative distribution was constructed from the histograms. By error function fitting analysis of the cumulative distributions (Figure S2), the averaged diffusion coefficients were estimated and summarized in Table 1. The averaged diffusion coefficient, 6.2 ± 1.1 μm^2^/s of EGFP-hGR wild type, was comparable to those previously reported at 8.7 ± 2.5 μm^2^/s by FCS [19] and 3.4 ± 1.0 μm^2^/s by FRAP [17]. By statistical analysis (student *t*-test), all diffusion coefficients were significantly different from each other (*p* < 0.001), except the pairs consisting of wild type-A458T, wild type-D196Y, and A458T-D196Y (Table S1).

### 2.2. Relationship between the Diffusion Property and Transcriptional Activities of GR Wild Type and Mutants

**Figure 4:**
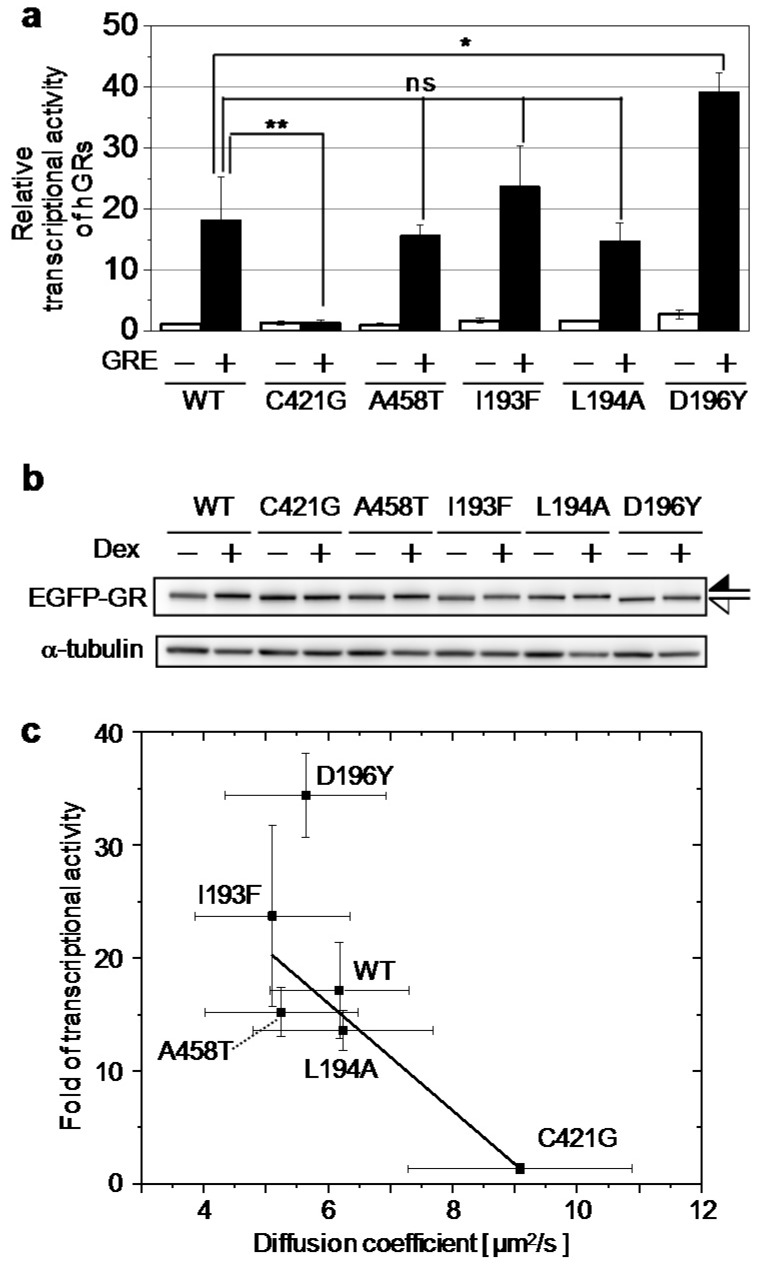
The relationship between the transcriptional activity and the diffusion coefficients of hGRs. (**a**) The relative transcriptional activities of hGR wild type and mutants were estimated by a luciferase assay using pGL4 (without GRE) and pGL4-GRE shown as open and solid bars, respectively. The relative transcriptional activity was calculated so that the normalized luciferase activity with an addition of 100 nM Dex was divided by the normalized luciferase activity with DMSO only. The mean and SD of fold transcriptional activity were obtained from three individual experiments. The transcriptional activity of EGFP-hGR^C421G^ and EGFP-hGR^D196Y^ were significantly smaller and larger than that of EGFP-hGR^WT^, respectively. *: *p* < 0.05, **: *p* < 0.001, ns: no significant differences (Student *t*-test); (**b**) the expression levels of EGFP-hGR wild type and mutants were estimated by a Western blot using anti-EGFP and anti-β-tubulin antibodies. The black arrow indicates the band shift of EGFP-hGR wild type and mutants except EGFP-hGR^D196Y^. The white arrow indicates the band shift of EGFP-hGR^D196Y^; (**c**) the relationship between the diffusion coefficients (from RICS) and the transcriptional activities (from the luciferase assay) of hGR wild type and mutants. The error bars on the *x*- and *y*-axis indicate the standard deviation (SD) and standard errors (SEM), respectively. The solid line indicates the linear regression by least squares method, adjusted *R*^2^ = 0.82.

To confirm the relationship between the diffusion property and transcriptional activities of hGR wild type and mutants, the transcriptional activities of hGR wild type and mutants were compared by luciferase assays. The transcriptional activity of EGFP-hGR^WT^ was increased 18.2 ± 7.0-fold by stimulation with Dex and co-transfection with a reporter plasmid containing GRE (Figure 4a, black bar). In contrast, the transcriptional activity of EGFP-hGR^C421G^ impaired DNA binding and was not changed by stimulation with Dex. Furthermore, the transcriptional activity of EGFP-hGR^C421G^ was significantly lower than that of wild type and other mutants. Mutants EGFP-hGR^A458T^ and EGFP-hGR^L194A^ impaired dimerization and transcriptional activity, respectively, and had slightly lower transcriptional activities than that of the wild type. In comparison, the transcriptional activities of EGFP-hGR^I193F^ and EGFP-hGR^D196Y^, which enhanced transcriptional activities, were higher than those of the wild type, as expected. The transcriptional activities were summarized in Table 2, and it was confirmed that expression levels of EGFP-hGR wild type and mutants were similar in the absence and presence of Dex (Figure 4b).

As shown in Figure 4c, the transcriptional activities were strongly and negatively correlated (adjusted *R*^2^ = 0.82) with the averaged diffusion coefficients estimated from RICS. This result shows that the diffusion property of hGR was directly related its transcriptional activity in a live cell nucleus. Interestingly, with regression between the transcriptional activities and the diffusion coefficients without EGFP-hGR^D196Y^, the adjusted *R*^2^ value was improved to 0.97 (Figure S3, red line). This result suggests that the mutant EGFP-hGR^D196Y^ had higher transcriptional activity than the expected relationship to the diffusion coefficients of the wild type and other mutants.

### 2.3. Phosphorylation State of GR Wild Type and Mutant

**Figure 5:**
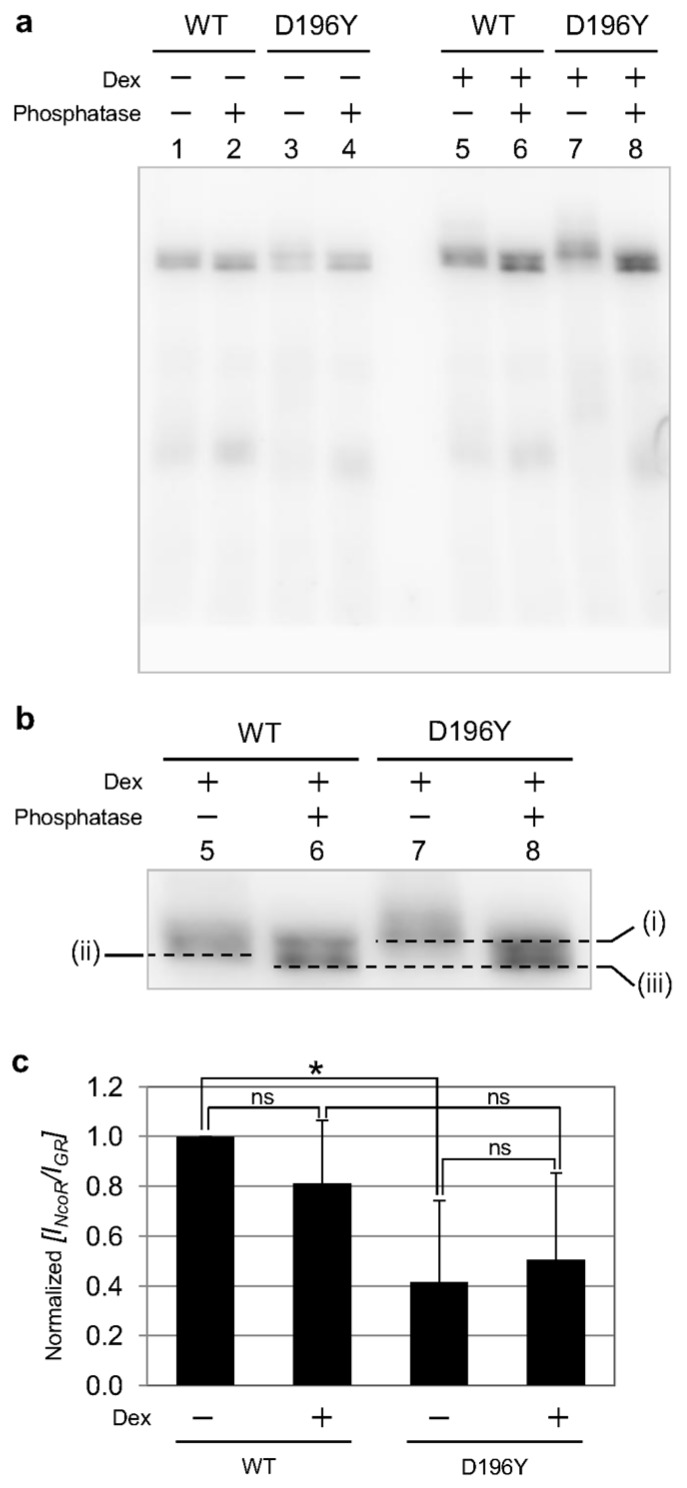
The differences between the hGR wild type and D196Y mutant for phosphorylation state and binding to NCoR. (**a**) Phos-tag PAGE for phosphorylation of EGFP-hGR^WT^ and EGFP-hGR^D196Y^. In the absence of Dex (lane 1–4), any differences in the band-shifts were not observed; (**b**) the enlarged image of lane 5–8 of (**a**) after stimulation with Dex. The band of EGFP-hGR^WT^ (from lane 5 to 6) and EGFP-hGR^D196Y^ (from lane 7 to 8) was shifted by the addition of the phosphatase. However, the band shift caused by the dephosphorylation of EGFP-hGR^D196Y^, corresponding to the shift from (i) to (iii), was greater than that of EGFP-hGR^WT^, corresponding the shift from (ii) to (iii); (**c**) binding of NCoR with hGR wild type and D196Y mutant was analyzed by coimmunoprecipitation. The tendencies of binding NCoR with the hGR wild type and D196Y mutant were shown as normalized to the relative intensity [*I_NCoR_/I_GR_*] of the bands quantified from Figure S5a,b. The mean and SD of normalized relative intensity [*I_NCoR_/I_GR_*] was obtained from three individual experiments. *: *p* < 0.05, ns: no significant differences. (Student *t*-test).

As shown in Figure 4b (upper) and Figure S4, the mobilities of bands corresponding EGFP-hGR^D196Y^ (white arrow) shifted slightly more than those of EGFP-hGR wild type and other mutants (black arrow). By combining the results of this shift and higher transcriptional activity of EGFP-hGR^D196Y^, hyper-phosphorylation of EGFP-hGR^D196Y^ was suggested because phosphorylation could affect the transcriptional activity of GR. Therefore, the phosphorylation state of EGFP-hGR^WT^ and EGFP-hGR^D196Y^ was analyzed by Phos-tag electrophoresis (Phos-tag PAGE). As shown in Figure 5a, lanes 1–4, in the absence of Dex, similar mobility for bands was observed for EGFP-hGR^WT^ and EGFP-hGR^D196Y^ both in the presence and absence of the phosphatase. This result shows that both EGFP-hGR^WT^ and EGFP-hGR^D196Y^ were not phosphorylated before stimulation by Dex. Conversely, after stimulation with Dex as shown in Figure 5b, the bands for both EGFP-hGR^WT^ and EGFP-hGR^D196Y^ were shifted by addition of a phosphatase (from lane 5 to 6 and from lane 7 to 8, respectively). Moreover, the band shift caused by dephosphorylation of EGFP-hGR^D196Y^ corresponding to the shift from (i) to (iii) was greater than that of EGFP-hGR^WT^ corresponding to the shift from (ii) to (iii). This result indicates that EGFP-hGR^D196Y^ was hyper-phosphorylated compared to EGFP-hGR^WT^.

The phosphorylation of GR controls its transcriptional activity by modifying cofactor interactions [10,32]. Thus, the interaction of EGFP-hGR^WT^ and EGFP-hGR^D196Y^ with nuclear corepressor (NCoR) was analyzed by the coimmunoprecipitation method. From the result of Western blotting using anti-NCoR and anti-GR, the amount of NCoR that interacted with EGFP-hGR^WT^ and EGFP-hGR^D196Y^ was quantified by relative band intensity (Figure 5c and Figure S5). The amount of NCoR that interacted with EGFP-hGR^WT^ was slightly decreased by Dex stimulation. Compared to the wild type, the amount of NCoR interacting with EGFP-hGR^D196Y^ was reduced in the absence and presence of Dex. This result suggests that the high transcriptional activity of EGFP-hGR^D196Y^ originated from impairing the interaction with the repressor NCoR by the hyper-phosphorylation of EGFP-hGR^D196Y^.

## 3. Discussion

The spatial distribution maps of the diffusion coefficients of EGFP-hGRs were constructed by RICS analysis in a living cell nucleus stimulated with Dex. As shown in Figure 2e, the diffusion coefficients of EGFP-hGR^WT^ were heterogeneously distributed in the nucleus after addition of Dex. This heterogeneity of EGFP-hGR^WT^ suggested that the crowding effect of GR and/or the functions of GR, such as direct or indirect interaction with DNA, was localized in nuclear subdomains. To identify the origin of heterogeneity in diffusion properties, EGFP-hGR mutants whose higher, lower, and no transcriptional activities were compared with wild type, and their averaged diffusion coefficients were determined by the error function fitting of the cumulative distribution, which was constructed from the diffusion histograms (Figure 3b,c).

For fitting analysis to calculate the diffusion coefficients of EGFP-hGR wild type and mutants in each brick, the lateral and axial radius of the PSF (*w*_0_ and *w*_z_) were determined to be 0.233 and 1.054 μm, respectively, by RICS analysis of EGFP-tetramers [33] in the cell nucleus because the molecular weight of the EGFP-tetramer is close to that of EGFP-hGR. In this study, using an EGFP-tetramer rather than Rhodamine 6G as a standard for the diffusion coefficient led to a good fitting result in this study, although the Rhodamine 6G is usually used as the standard material (diffusion coefficient = 414 μm^2^/s [34]) for calculation of the absolute diffusion coefficient in diffusion analysis, such as RICS and FCS.

By RICS analysis, we demonstrated that the diffusion coefficients were strongly and negatively correlated with their transcriptional activities (Figure 4c). This result suggests that the decrease in the diffusion coefficient contributed to the high-affinity interaction between hGR and GBR rather than tethering to other transcription factors, such as NF-κB because the diffusion coefficient of hGRs was not correlated (the *R*^2^ value was negative) with the transrepressional activity to NF-κB (Figure S6). However, our RICS analysis could not clearly distinguish the hGR wild type and mutants as diffusion coefficient, with exception of C421G. Two-component fitting analysis of spatial correlations was suggested as the preferred method to improve quantification of the diffusion property of hGRs because the diffusion coefficient of hGR wild type and A458T mutant could be distinguished by two-component fitting analysis in our previous report using FCS [19]. Unfortunately, the measurement condition in this study was not sufficient for two-component fitting analysis, because of low signal-to-noise from EGFP labeled hGRs. Therefore, improving the fluorescence material and detectors could be used to analyze the diffusion properties among the hGR wild type and mutants in the future. Furthermore, to focus the slow mobility component that originated from directly binding of hGR to GBR, not just the RICS analysis but also in combination with the temporal image correlation spectroscopy (TICS) [17,35] and single plane illumination fluorescence correlation spectroscopy (SPIM-FCS) [36] may be required.

**Figure 6:**
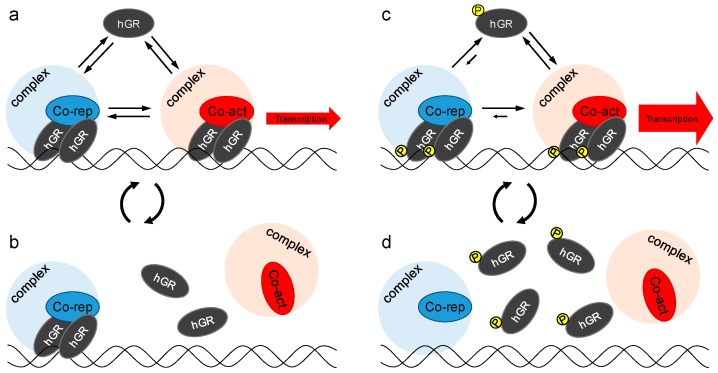
The model of effect of hyper-phosphorylation on transcriptional activity and mobility of hGR. Transcriptional activation is initiated by binding hGR to GBR but controlled by association with the coactivator and corepressor complex. The observed diffusion coefficient was reflected in the total states of hGR. (**a**,**b**) Restricting the mobility of hGR by association with the corepressor complex resulted in a lower diffusion coefficient; (**c**,**d**) hyper-phosphorylation of hGR reduced association with the corepressor complex; thus, the transcriptional activity and diffusion coefficient were increased. Co-rep (blue) and Co-act (red) represent the corepressors (such as NCoR) and coactivators (such as GRIP1), respectively. The light blue and red circles are the complex of corepressor and coactivator, respectively. The hGR with a “P” in yellow circle on it represents hyper-phosphorylation.

Moreover, by analyzing the relationship between the diffusion coefficients and the transcriptional activity, the D196Y mutant seemed to be ostracized from the group of the hGR wild type and other mutants. Then we found that the hyper-phosphorylation of EGFP-hGR^D196Y^ (Figure 5a,b) enhanced its transcriptional activity by diminishing its association with NCoR, a nuclear corepressor (Figure 5c). This hyper-phosphorylation of EGFP-hGR^D196Y^ was not affected by the transrepressional activity to NF-κB (Figure S6a). These results suggest that restricting the mobility of hGR by tethering it to GBR is necessary for transcriptional activation by hGR (Figure 6a,b), but hyper-phosphorylation of hGR makes turnover of the hGR-GBR interaction faster by reducing the association of NCoR with hGR (Figure 6c,d). However, whether this phosphorylation could cooperatively enhance or reduce phosphorylation of other amino acid, such as S203, S211, and S226, was still unknown although our results suggest that the mutated tyrosine on amino acid 196 of hGR may be phosphorylated.

Finally, we concluded that the diffusion coefficient obtained by diffusion analysis using RICS was directly reflected in the transcriptional activity and noninvasively predicted the hyper-phosphorylation in hGR live cells. Thus, we proposed that the spatial distribution of the transcriptional activity could be noninvasively and continuously estimated with a duration of a few tens of seconds by analysis of diffusion coefficients obtained from RICS.

## 4. Materials and Methods

### 4.1. Chemicals and Antibodies

Dexamethasone was purchased from SIGMA-Aldrich (St. Louis, MO, USA) and used in DMSO (dimethyl sulfoxide) solutions. The antibody anti-EGFP (GF200) was purchased from Nacalai tesque (Kyoto, Japan), anti-α-tubulin (clone DM1A, #05-829) from EMD Millipore (Billerica, MA, USA), anti-NCoR (sc-8996) and anti-mouse-HRP (sc-2031) were from Santa Cruz Biotechnology, Inc. (Dallas, TX, USA), and anti-rabbit-HRP (111-035-144) from Jackson ImmunoResearch Inc. (West Grove, PA, USA).

### 4.2. Cell Culture and Transient Transfection

The U2OS cells were maintained in a 5% CO_2_ humidified atmosphere at 37 °C in the McCoy’s 5A modified medium supplemented with 10% GIBCO charcoal-stripped fetal bovine serum (Thermo Fisher Scientific, Waltham, MA, USA). One day before transfection, 0.5 × 10^5^ U2OS cells were subcultured in an Nunc 8-well chambered slide (Thermo Fisher Scientific, Waltham, MA, USA). For RICS and FCS measurement, U2OS cells were transfected using the lipofection reagent Optifect (Thermo Fisher Scientific, Waltham, MA, USA) 1 μL/well and 0.1 μg/well pEGFP-tetramer, pEGFP-hGR^WT^ (wild type), pEGFP-hGR^C421G^, pEGFP-hGR^A458T^, pEGFP-hGR^I193F^, pEGFP-hGR^L194A^, or pEGFP-hGR^D196Y^ with 0.1 μg/well pH2B-mCherry and pTagBFP-fibrillarin. H2B-mCherry and TagBFP-fibrillarin were expressed as the markers for the nucleus and nucleolus, respectively.

### 4.3. Raster Image Correlation Spectroscopy (RICS)

For RICS analysis, LSM images were collected by LSM 710 META ConfoCor3 (Carl Zeiss, Oberkochen, Germany). The excitation light was directed to the sample by a dichroic mirror (HFT 488/543) and a C-Apochromat 40×/NA 1.2 water immersion objective. The emission was detected by APD through the band-pass BP505-610 emission filter. A pinhole was set at 1 airy unit (39 μm). The scan speed was 3.15 μs/pixel and 1.62 ms/line. The image size was set to 256 × 256 pixels, and the zoom factor was set to 40 (*δ_r_* = 21 nm) to ensure that the PSF contained a sufficiently large number of pixels (radius of about 10 pixels) and that the region for RICS had enough spatial resolution to avoid the region of a nucleolus.

Two-dimensional correlation analysis of a temporal series of LSM images was performed by a laboratory-made software made in MATLAB R2015b (Math Works, Natick, MA, USA) with the Optimization Toolbox. Fluorescence intensity in LSM images usually contained an immobile component which prevents RICS analysis of the mobile component especially in the case of cell analysis. To remove the immobile component in the LSM images, a detrend processing [23,27] was performed. The detrended image *I*′ of the *n*_0_-th raw image *I* in a time-stack image can be expressed as follows:

(1) $$I^{\prime }(x,y,n_{0})=I(x,y,n_{0})-\bar{I_{t}}(x,y)+\bar{I_{s}}(n_{0})$$

(2) $$\bar{I_{t}}(x,y)=\frac{1}{2d+1}\sum_{n=n_{0}-d}^{n_{0}+d}I(x,y,n)$$

where *x* and *y* are the spatial coordinate of the image, and *n* is the frame number in the time-stack image. The time averaged image $\bar{I_{t}}$ of (2*d* + 1) frames is subtracted from raw image, and the spatial average of entire *n*_0_-th raw image $\bar{I_{s}}\left(n_{0}\right)$ is added. It is important that once the average fluorescence intensity is changed by detrend processing, the amplitude of the autocorrelation function is no longer corresponding to *1/N* [23].

An analysis windows with the size of 64 × 64 pixel and a step size of 32 pixel (half of analysis window was overlapped) were applied along all 100 frames of 256 × 256 pixel, and the two-dimensional auto-correlation functions were calculated in each analysis window. As a result, 7 × 7 auto-correlation functions were obtained from each frame of the image stack. Finally, a temporally averaged 7 × 7 auto-correlation functions were obtained.

In the free diffusion condition, the auto-correlation function *G*(*ξ*, *η*) can be represented as follows using the laser scanning term *S_n_* and the diffusion term *G_D,n_* for the *n*-th diffusion species:

(3) $$G(\xi ,\eta )=\sum_{n}F_{n}\cdot S_{n}(\xi ,\eta )\cdot G_{D,n}(\xi ,\eta )$$

(4) $$S_{n}(\xi ,\psi )=\exp\left[-\frac{{\left(\frac{\xi \delta_{r}}{w_{0}}\right)}^{2}+{\left(\frac{\psi \delta_{r}}{w_{0}}\right)}^{2}}{1+\frac{4D_{n}\left(\tau_{p}\xi +\tau_{l}\psi \right)}{w_{0}{}^{2}}}\right]$$

(5) $$G_{D,n}(\xi ,\psi )=\frac{\gamma }{N}{\left[1+\frac{4D_{n}\left(\tau_{p}\xi +\tau_{l}\psi \right)}{w_{0}{}^{2}}\right]}^{-1}{\left[1+\frac{4D_{n}\left(\tau_{p}\xi +\tau_{l}\psi \right)}{w_{z}{}^{2}}\right]}^{-1/2}$$

where *ξ* and *η* are pixel shifts in *x*- and *y*-direction, respectively. *δ_r_*, *w*_0_, *w_z_*, *τ_p_*, and *τ_l_* represent the pixel size, lateral radius of confocal volume, axial radius of the volume, pixel dwell time, and line time, respectively. *N* is the average number of molecules of interest. *F_n_* and *D_n_* are the fraction and the diffusion coefficient of *n*-th species, respectively. Summation of all fractions should be 1.0. γ = 0.35 is the correction factor for a three-dimensional Gaussian illumination profile of laser illumination.

To extract the parameter of dynamics from the auto-correlation functions, non-linear least squares were conducted on each auto-correlation function in the averaged 7 × 7 auto-correlation function by minimizing the reduced χ^2^. In the analysis, only the *ξ*- and *η*-cross section of the auto-correlation function, *G*(*ξ*, 0) and *G*(0, *η*), were used sharing the parameters to be optimized. To the eliminate the noise component due to shot, after pulsing, and other noises, the data points around the origin (|ξ|≤2 or |η|≤2) were excluded from the least squares analysis. The lateral and axial radii of the PSF, respectively, *w*_0_ = 0.233 μm and *w_z_* = 1.054 μm, were used as fixed parameters. The axial radius, *w_z_* = 1.054 was determined by FCS measurements of rhodamine 6G solution. The lateral radius, *w*_0_ = 0.233 μm was determined by RICS measurements of EGFP-tetramer expressing cells by fixing the axial at *w_z_* = 1.054 μm. As a result of the fitting analysis, the distributions of the diffusion coefficient or other parameters were generated by mapping the fitted parameters.

The diffusion coefficient histograms were constructed by integrating the numbers calculated by one-component fitting analysis of each brick in the diffusion map which was obtained from measurements of 6–8 cells. The fitting of unsuccessful data of bricks in diffusion maps reduced χ^2^ by more than 1.05, and the data of heavily expressing cells whose number of molecule were calculated as than 20 were excluded from the histograms.

The cumulative distributions that were constructed from the diffusion coefficient histograms were fitted by Equation (6):

(6) $$y=\frac{1+erf\left(\frac{x-D}{\sqrt{2}\cdot s}\right)}{2}$$

where *erf*(*x*) indicate an error function, *D* and *s* are the average and a standard deviation of diffusion coefficients, respectively.

### 4.4. Luciferase Assay for Transcriptional Activity of hGR

A day before transfection, 1 × 10^6^ U2OS cells were seeded on a Nunc 6-well plate (Thermo Fisher Scientific, Waltham, MA, USA). The U2OS cells were transfected using the lipofection reagent Lipofectamine2000 (Thermo Fisher Scientific, Waltham, MA, USA) with 10 μL/well and 1.5 μg/well for pEGFP-hGR^WT^, pEGFP-hGR^C421G^, pEGFP-hGR^A458T^, pEGFP-hGR^I193F^, pEGFP-hGR^L194A^, or pEGFP-hGR^D196Y^ and with 1.5 μg/well pGL4 (Promega, Wisconsin, WI, USA) or pGL4-GRE as a reporter. pRL-CMV (Promega, Wisconsin, WI, USA) was the internal control. Twenty-four hours after transfection, 100 nM Dex (or DMSO) was added. Six hours after addition of Dex, U2OS cells were trypsinized and harvested in 1.5 mL micro tubes. After washing with cold PBS supplemented with 0.8 mM AEBSF (4-(2-aminoethyl)benzenesulfonyl fluoride hydrochloride), luciferase assay was performed using Dual-Glo Luciferase Assay System (Promega, Wisconsin, WI, USA) according to the manufacturer’s instructions. The chemiluminescence from the firefly and renilla luciferase was measured and analyzed by Typhoon TRIO + Variable mode imager (GE healthcare, Little Chalfont, UK) and ImageQuant TL software (GE healthcare, Little Chalfont, UK). The activity of firefly luciferase which was evoked by activation of hGR was normalized by the activity of renilla luciferase using the following Equation (7):

(7) $$\mathrm{Normalized}\;\mathrm{luciferase}\;\mathrm{activity}=\frac{I_{f}-I_{f,background}}{I_{r}-I_{r,background}}$$

where *I_f_* is the intensity of firefly luciferase, *I_r_* is the intensity of renilla luciferase, and *I_f,background_* and *I_r,background_* are background intensity of firefly and renilla luciferase activity, respectively. Then, the relative transcriptional activity was calculated from the normalized luciferase activity where the 100 nM Dex addition sample was divided by the normalized luciferase activity from DMSO only. The mean and SD of transcriptional activities were calculated from three individual luciferase assays.

### 4.5. Phos-Tag PAGE

A day before transfection, 5 × 10^5^ U2OS cells were seeded on a Nunc 6-well plate (Thermo Fisher Scientific, Waltham, MA, USA). The U2OS cells were transfected using 6 μL/dish lipofection reagent Viafect (Promega, Wisconsin, WI, USA) and 3 μg/dish pEGFP-hGR^WT^ and pEGFP-hGR^D196Y^. Twenty-four hours after transfection, 100 nM Dex (or DMSO) was added. Six hours after addition of Dex, U2OS cells were washed by PBS and trypsinized. Cells were collected in 1.5 mL microtubes and washed by PBS supplemented 0.8 mM AEBSF. After centrifugation at 6000× *g* for 5 min at 4 °C, cells were lysed in 25 μL lysis buffer, CelLytic M (SIGMA-Aldrich, St. Louis, MO, USA) supplemented, 1% (*v*/*v*) protease inhibitor cocktail (SIGMA-Aldrich, St. Louis, MO, USA), 1% (*v*/*v*) phosphatase inhibitor cocktail (Nacalai tesque, Kyoto, Japan), 0.5% SDS, and 2 U/μL Benzonase (SIGMA-Aldrich, St. Louis, MO, USA) for 30 min on ice. After centrifugation (17,400× *g*, 10 min, 4 °C), the cell lysate was recovered in the supernatant. The protein concentration of cell lysate was determined using the Bradford Ultra reagent (Novexin Ltd., Cambridge, UK), and concentrations were adjusted by dilution. Using a part of the cell lysate, dephosphorylation was performed by 20 U/μL Lambda Protein Phosphatase (LPP, New England Biolabs Inc., Ipswich, MA, USA) supplemented with 2 mM MnCl_2_ for 60 min at 30 °C.

For detection of the phosphorylated hGR wild type and mutant, Phos-tag PAGE was performed. The 5% polyacrylamide gel containing 50 μM acrylamide-pendant Phos-tag (Wako Pure Chemical Industries, Ltd., Tokyo, Japan) was made according to the manufacturer’s instructions. The electrophoresis was performed at 20 mA/gels for 120 min using a Phos-tag gel. In this electric current condition, the electrophoresis usually should be finished within 90 min. To improve the separation of bands, the time of electrophoresis was extended to 120 min in this experiment. After electrophoresis, the gel was washed in transfer buffer (48 mM Tris, 39 mM glycine, 10% methanol, and 0.0375% SDS) with 10 mM EDTA (ethylenediaminetetraacetic acid) for 10 min 3 times, and then without EDTA for 10 min. Proteins were transferred onto an Immun-Blot PVDF membrane (Bio-Rad, Hercules, CA, USA), and membranes were blocked in PBS containing 5% (*w*/*v*) skim milk and 0.05% (*v*/*v*) Tween 20. After incubation with the anti-EGFP antibody (GF200) in CanGetSignal solution 1 (TOYOBO CO., Ltd, Osaka, Japan), membranes were incubated with anti-mouse IgG conjugated with horseradish peroxidase in CanGetSignal solution 2 (TOYOBO CO., Ltd., Osaka, Japan). Specific binding of antibodies was imaged by LAS4000mini (Fujifilm corporation, Tokyo, Japan) using ECL Western Blotting Detection System (GE healthcare, Little Chalfont, UK) as a chemiluminescence.

## Figures and Tables

**Table 1:** Diffusion coefficients of hGR wild type and mutants.

	Wild Type	C421G	A458T	I193F	L194A	D196Y
Diffusion Coefficient (μm^2^/s)	6.2 ± 1.1	9.1 ± 1.8	5.2 ± 1.2	5.1 ± 1.2	6.2 ± 1.4	5.6 ± 1.3

Mean ± SD.

**Table 2:** Transcriptional activities of hGR wild type and mutants.

	Wild Type	C421G	A458T	I193F	L194A	D196Y
Fold of Transcriptional Activity	17 ± 4	1.3 ± 0.4	15 ± 2	23 ± 8	13 ± 1	34 ± 3

Mean ± SEM.

## Supplementary Materials

Supplementary Materials can be found at www.mdpi.com/1422-0067/18/9/1855/s1.
