# Supplementary material for: Negative Correlation between the Diffusion Coefficient and Transcriptional Activity of the Glucocorticoid Receptor
Source: Int J Mol Sci. 2017 Aug 25;18(9):1855. doi: 10.3390/ijms18091855 (PMC5618504; doi:10.3390/ijms18091855)
Supplement: Supplementary file 1 [file ijms-18-01855-s001.pdf]

Supplementary information

Negative correlation between the diffusion coefficient and transcriptional activity of the glucocorticoid receptor

Shintaro Mikuni, Johtaro Yamamoto, Takashi Horio, and Masataka Kinjo

Supplementary Figure S1

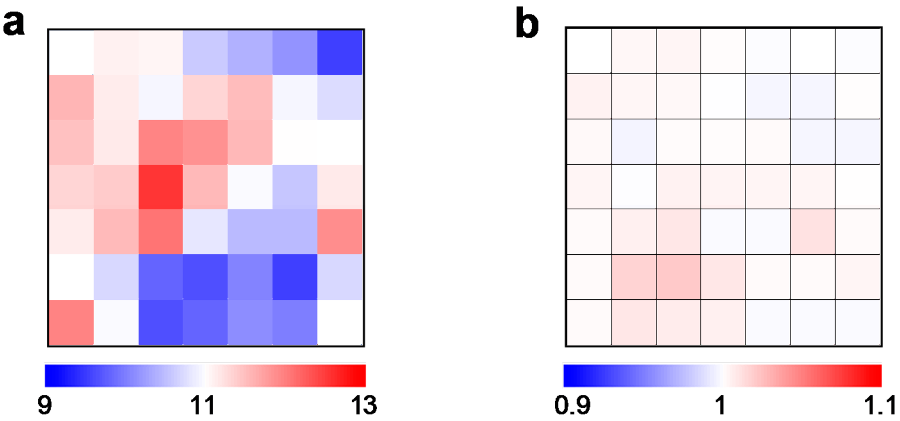

Supplementary Figure S1. The maps of numbers and reduced χ^2. The 7 × 7 map of (**a**) numbers and (**b**) reduced χ^2 in the region represented in Figure 2a.

Supplementary Figure S2

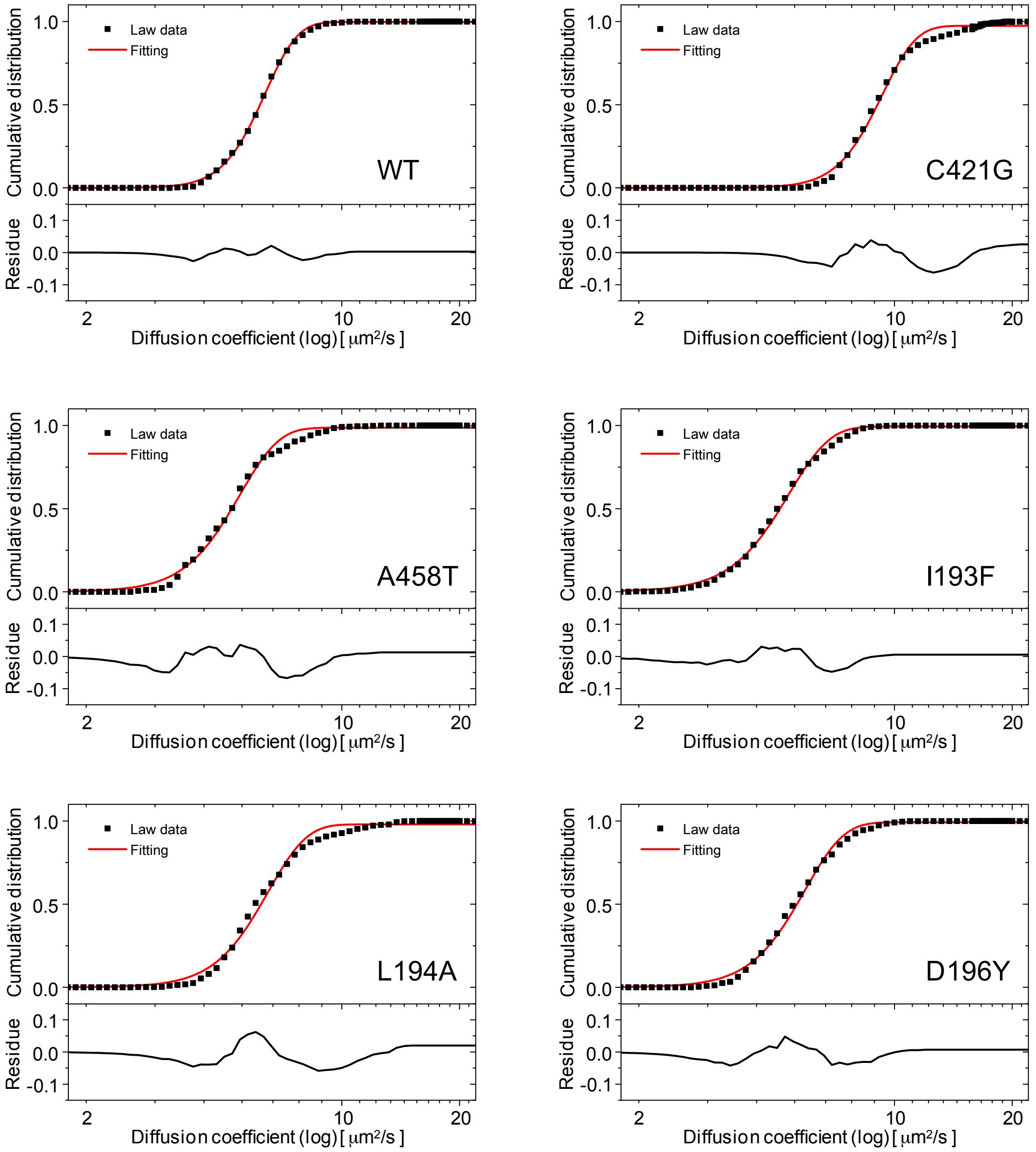

Supplementary Figure S2. The error function fitting of the cumulative distribution of hGR wild type and mutants. The black points and red lines indicate the raw cumulative distributions and the fitted error functions, respectively, in the upper panels. The black lines represent the residues in lower panels.

Supplementary Table S1

Supplementary Table 1. Statistical analysis of diffusion coefficients.

	Wild type	C421G	A458T	I193F	L194A	D196Y
Wild type	-	*	ns	*	*	ns
C421G	-	-	*	*	*	*
A458T	-	-	-	*	*	*
I193F	-	-	-	-	*	ns
L194A	-	-	-	-	-	*
D196Y	-	-	-	-	-	-

*: P<0.001, ns: no significant

Supplementary Figure S3

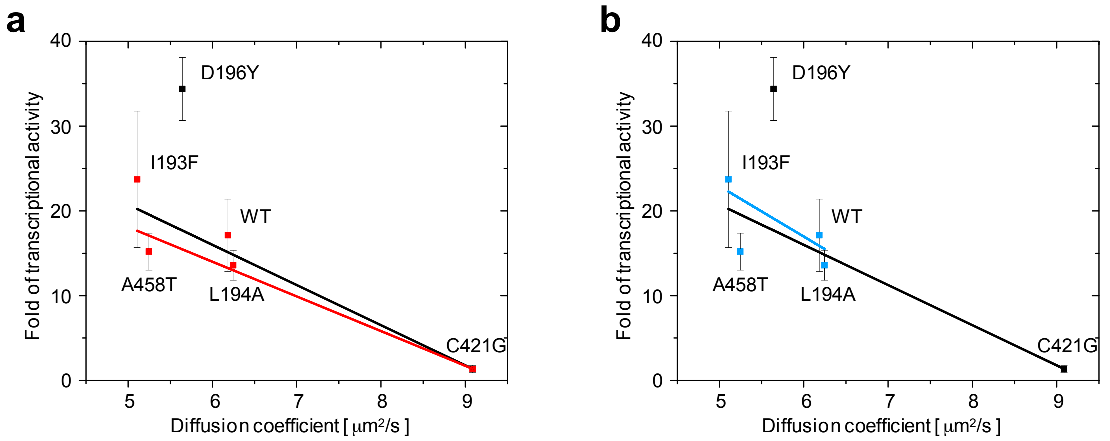

Supplementary Figure S3. The relationship between the averaged diffusion coefficients (from RICS) and the transcriptional activities from the luciferase assays of hGR wild type and mutants except EGFP-hGR^{D196Y} (a) or except EGFP-hGR^{C421G} and EGFP-hGR^{D196Y}; (b). The black line indicates the linear regression between the diffusion coefficients and transcriptional activities of the hGR wild type and mutants by the least squares method, adjusted $R^2 = 0.82$. The error bars indicate the standard errors. (a) The red points and line indicate the linear regression without the data of EGFP-hGR^{D196Y} (as shown by black point). The adjusted R^2 value was improved to 0.98. (b) The blue points and line indicate the linear regression without data of EGFP-hGR^{C421G} and EGFP-hGR^{D196Y} (as shown by the black points). The adjusted R^2 value was 0.36, a weak negative correlation.

Supplementary Figure S4

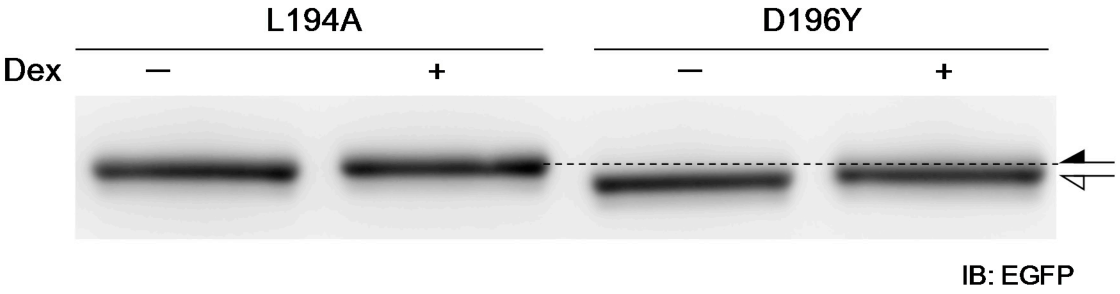

Supplementary Figure S4. The enlarged image of Figure 4b. The black arrow indicates the band shift of EGFP-hGR wild type and mutants except EGFP-hGR^{D196Y}. The white arrow indicates the band shift of EGFP-hGR^{D196Y}.

Supplementary Figure S5

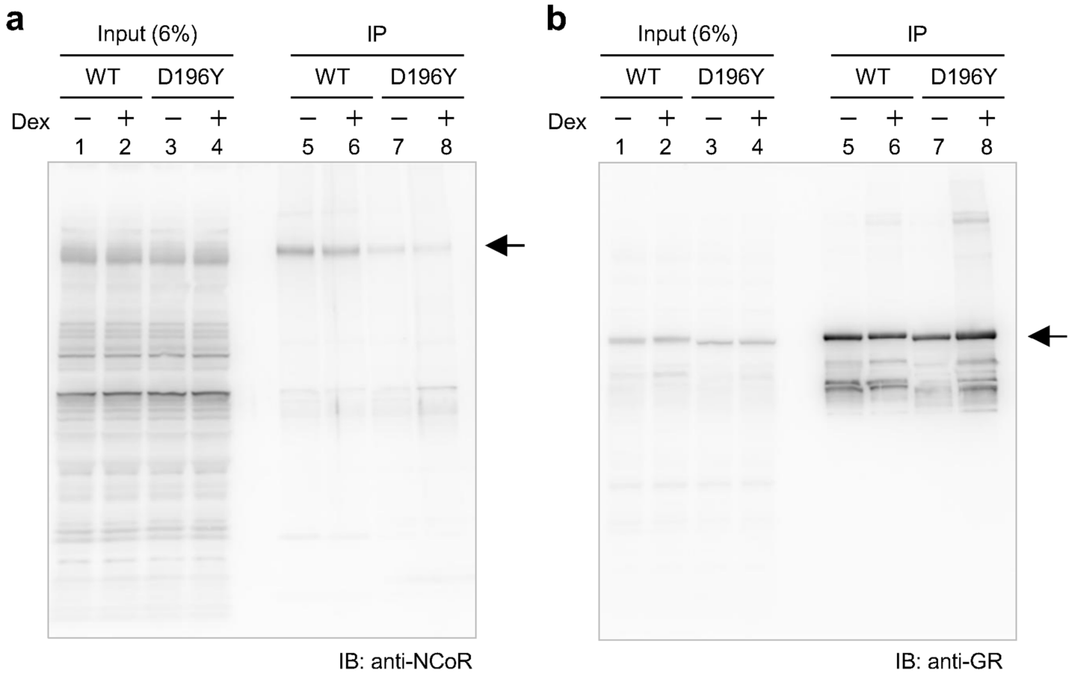

Supplementary Figure S5. Whole gel image shown in Figure 5c. The western blot analysis using (**a**) anti-NCoR and (**b**) anti-GR were shown. The solid arrow in (a) indicates the bands of NCoR (about 250 kDa) and the solid arrow in (b) indicates the bands of EGFP-hGR^{WT} and EGFP-hGR^{D196Y} (about 120 kDa).

Supplementary Figure S6

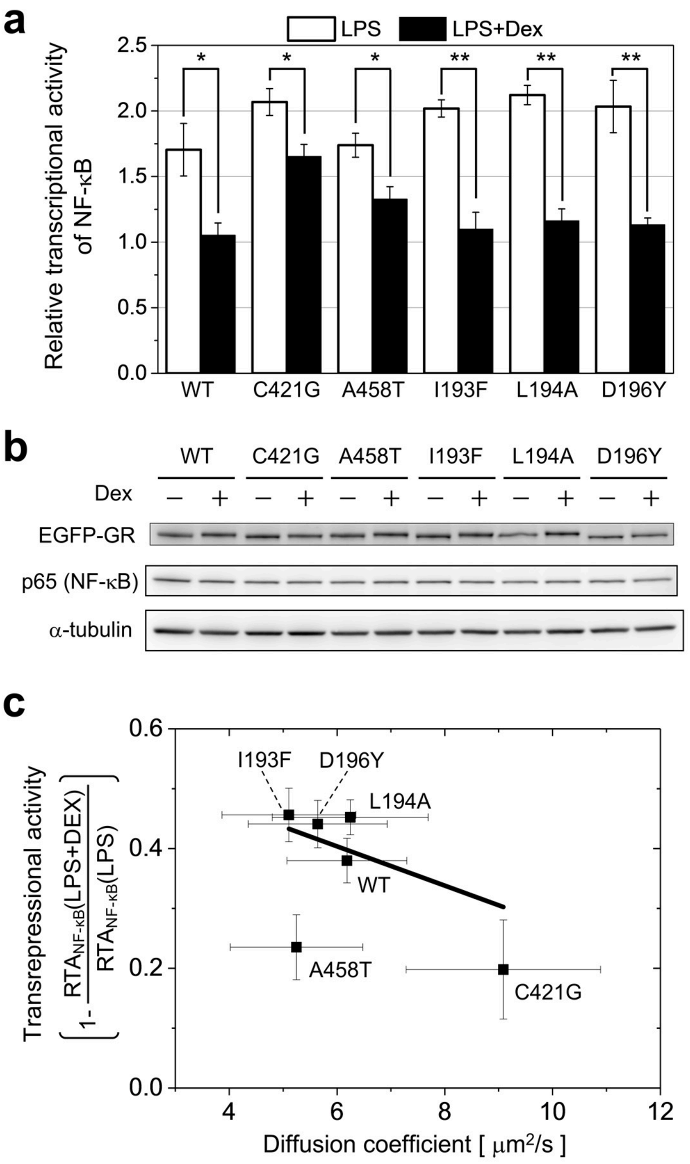

Supplementary Figure S6. The relationship between the transrepressional activities and the diffusion coefficients of EGFP-hGR wild type and mutants. (**a**) The relative transcriptional activities of NF-κB co-expressed with EGFP-GR wild type and mutants were estimated by a luciferase assay using pGL4-NF-κB-RE (response element). The open and solid bars represent the addition of 200 ng/mL LPS only and with 100 nM Dex, respectively. LPS was used as an activator of NF-κB. The relative transcriptional activities were calculated as division of luciferase activity by DMSO only. The mean and SD of relative transcriptional activities were obtained from three individual experiments. Addition of Dex significantly repressed the transcriptional activity of NF-κB when EGFP-hGR wild type and mutants were coexpressed. * $p < 0.001$, ** $p < 0.05$ (Student t-test). (**b**) The expression levels of EGFP-GR wild type and mutants and p65, which is a subunit of NF-κB, were checked by western blotting using anti-GR, anti-p65, and anti-β-tubulin antibodies. The similar expression levels of EGFP-GR wild type and mutants were observed. (**c**) The relationship between the diffusion coefficients and transrepressional activity of the GR wild type and mutants. RTA indicates relative transrepressional activity calculated from (a). The black line indicates the linear regression by the least squares method, the adjusted R^2 = -0.11.

Supplementary Figure S7

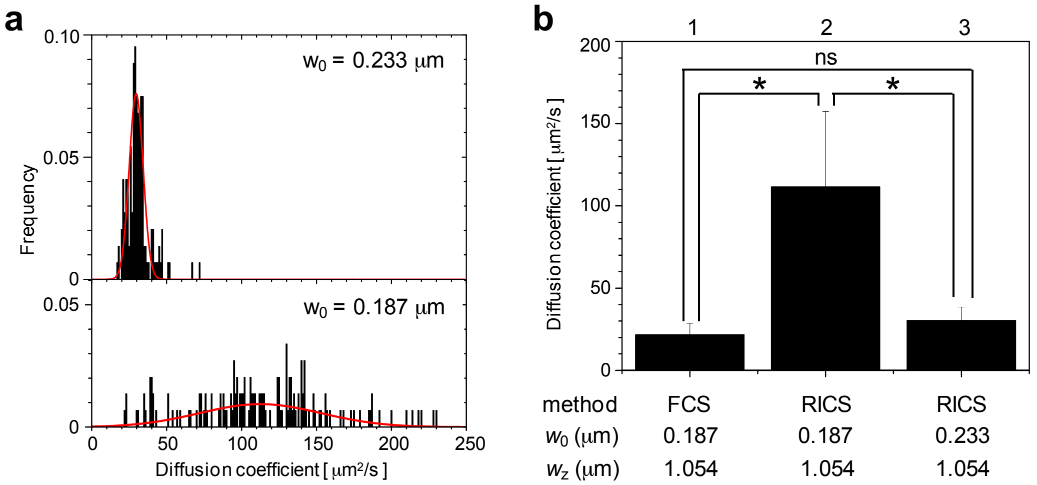

Supplementary Figure S7. Effect of structure parameter w_0 on diffusion coefficient in FCS and RICS. (**a**) The diffusion coefficient histograms of the EGFP-tetramer by RICS fitting analysis of the same images using w_0 = 0.233 μm (upper) and w_0 = 0.187 μm (lower). Y-axis represents the frequency of the number of bricks that were analyzed by RICS (n = 147 bricks). The red lines represent single Gaussian fitting. Adjusted R^2 = 0.84 and 0.21 in the upper and lower histogram, respectively. (**b**) The averaged diffusion coefficient of the EGFP-tetramer was calculated by the indicated method and the lateral and axial radius of PSF. * : $p < 0.05$, **: $p < 0.001$, ns: no significant differences (Student *t*-test).

Supplementary Figure S8 and Table S2

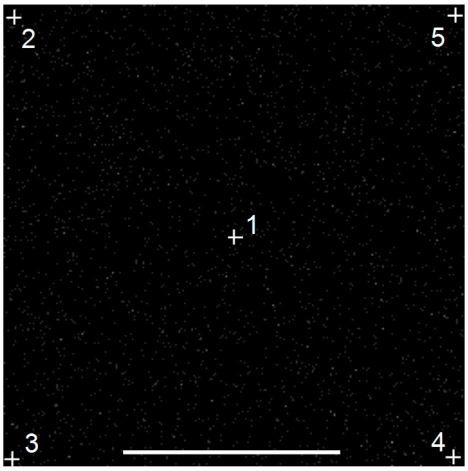

Supplementary Figure S8. LSM image of the rhodamine 6G solution. The white crosses indicate the FCS measurement position at the center (position 1) and the off-centers (position 2-5). Scale bar = 100 μm. The lateral and axial radius at positions 1-5 were summarized in Supplementary Table S1.

Supplementary Table S2.
The lateral and axial radius at the center and off-centers in FCS

	radius [μm]	
position	lateral (w_0)	axial (w_z)
1	0.179	1.115
2	0.180	1.525
3	0.186	1.295
4	0.188	1.041
5	0.191	0.940

Supplementary Figure S9

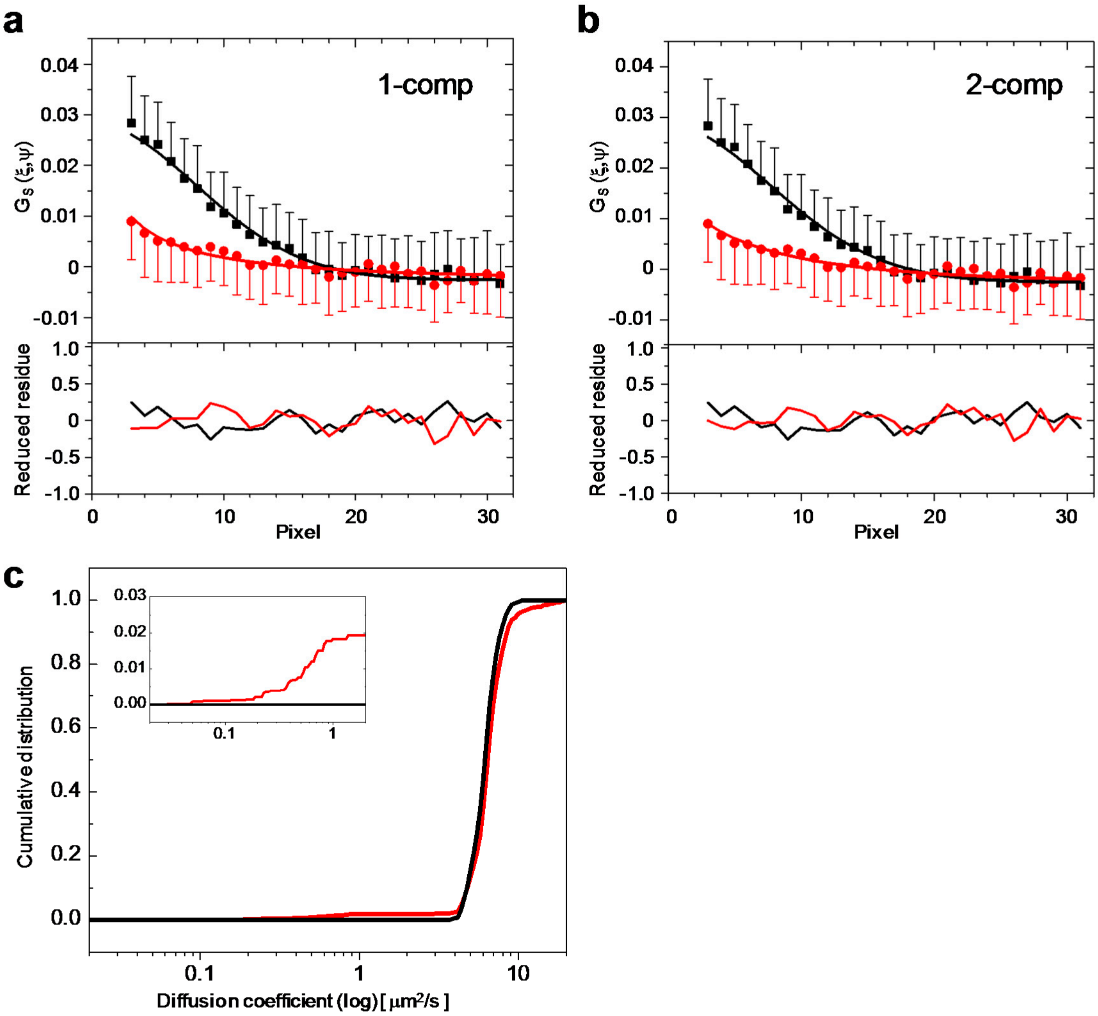

Supplementary Figure S9. One- and Two-component fitting for the spatial correlation. The spatial correlation and fitting with a (**a**) one-component or (**b**) two-component diffusion model (upper), and its reduced residue (lower) are indicated by an asterisk (*) in Figure 2e. The black and red points indicate the spatial correlation to the x- and y-direction of the images, respectively. (**c**) The cumulative distribution which was constructed from the diffusion coefficient histogram by one-component (black line) and two-component (red line) fitting model. Inset represents the enlarged range less than 2 $\mu m^2/s$.

Supplementary discussion

For fitting analysis to calculate the diffusion coefficients of the EGFP-hGR wild type and mutants in each brick, the lateral and axial radius of the PSF (w_0 and w_z) were determined to be 0.233 and 1.054 μm, respectively, by RICS analysis of EGFP-tetramer [1] in the cell nucleus because the molecular weight of EGFP-tetramer is close to that of EGFP-hGR. Using the parameters of PSF, the averaged diffusion coefficients of an EGFP-tetramer in the cell nucleus were calculated as a single Gaussian distribution (Supplementary Figure S7a, upper) by RICS, and its averaged diffusion coefficient was similar to that calculated from FCS (Supplementary Figure S7b, bar 1 and 3). However, the averaged diffusion coefficient of the EGFP-tetramer by RICS using w_0 = 0.187 μm and w_z = 1.054 μm, which was determined by FCS measurement of Rhodamine 6G (diffusion coefficient = 414 $\mu m^2/s$) [2], was larger than that calculated by FCS (Supplementary Figure S7b, bar 1 and 2), and their distribution appeared discrete and not Gaussian (Supplementary Figure S7a, lower). This discrepancy of diffusion coefficients may be caused by the PSF being deformed in scanning images at an off-center position of the focal plane for RICS because the PSF was usually defined by measurements at the center of the focal plane in FCS. As shown in Supplementary Figure S8 and Table S2, the PSF at an off-center (position 2-5 in Supplementary Figure S8) was deformed and slightly expanded compared with that at the center (position 1 in Supplementary Figure S8) of the focal plane. Moreover, it may be suggested that this deforming effect is increased in the case of living cell measurement.

The distribution of the diffusion coefficient of the EGFP-tetramer using w_0 = 0.187 μm was more discrete than that using w_0 = 0.233 μm (Supplementary Figure S7a). One of the reasons for this heterogeneity is thought to arise from low-accuracy fitting by using w_0 = 0.187 μm because the averaged χ^2 using w_0 = 0.187 μm (1.018 ± 0.013) was significantly larger than that using w_0 = 0.233 μm (1.004 ± 0.007). Therefore, the averaged lateral radius w_0 = 0.233 μm, which was determined by RICS analysis of EGFP-tetramer in living cells, was practically proper to analyze the diffusion coefficient of EGFP-hGRs.

In this study, the spatial correlation functions of all bricks were calculated from a 64x64 pixel of 100-frame images and successfully fitted with a one-component diffusion model although the functions had large errors. For generation of a diffusion map, the image area was divided into 7 × 7 bricks in this study. That is, each brick had 64x64 pixels per image. Therefore, the spatial correlation on each brick was calculated from a 64x64 (pixels/image) x100 compilation, approximately 4×10^6 pixels. We believe this pixel number was sufficient for calculation of spatial correlation, because the spatial correlation from 128x128x100 as trial calculation was not dramatically improved.

It is possible that this large error originated from the two-components for the two

reasons explained below. One, the reduced χ^2 value was not dramatically increased by changing the model from a one-component into a two-component (Supplementary Figure S9a, b). Two, the two-component-applicable data was less than 10% of all data. Thus, when the histogram was constructed by applying two-component fitting models, the cumulative distribution (Supplementary Figure S9c, red line) was not successfully fitted by the two-component error function because the slow component, whose diffusion coefficient is less than 1 $\mu m^2/s$, may be too small (less than 2% in the cumulative distribution) to estimate the diffusion coefficient by error function fitting. The slow mobility component of hGR is thought to originate from directly binding to GBR (GR binding regions), including GRE [3-11]. Therefore, to focus the slow mobility component of hGR as diffusion maps, not only RICS analysis but the combination with temporal image correlation spectroscopy (TICS) [9, 12] and single plane illumination fluorescence correlation spectroscopy (SPIM-FCS) [13] may be required.

Supplementary materials and methods

Antibodies

The anti-GR (ab3579) was purchased from Abcam PLC (Cambridge, UK).

Plasmid construction

The plasmids encoding EGFP fused hGR wild type (EGFP-hGR^{WT}) and mutants EGFP-hGR^{C421G} and EGFP-hGR^{A458T} were constructed as previously reported [11] [1]. The point mutants, EGFP-hGR^{I193F}, EGFP-hGR^{L194A}, and EGFP-hGR^{D196Y} were constructed by a two-step PCR procedure [11] with primers containing mutations. The combination of primers was summarized in Supplementary Table S3. After digestion of PCR products containing each mutation by Sal I and Cla I, the digested fragments were inserted into a pEGFP-hGR vector that was cut with the same restriction enzymes. All of the above PCRs were performed using KOD-Plus- (TOYOBO CO., Ltd, Osaka, Japan) according to the manufacturer's instructions.

Table S3: The primers for mutation of GR

Forward	I193F	: 5'-	ACCTTTGACttcTTGCAGGATTTG	-3'
Reverse	I193F	: 5'-	ATCCTGCAAgaaGTCAAAGGTG	-3'
Forward	L194A	: 5'-	TTTGACATTgccCAGGATTTGGA	-3'
Reverse	L194A	: 5'-	CAAATCCTGggcAATGTCAAAGGT	-3'
Forward	D196Y	: 5'-	ATTTTGCAGtatTTGGAGTTTTC	-3'
Reverse	D196Y	: 5'-	AAACTCCAAataCTGCAAAATG	-3'

*The lower-case indicates the nucleotides mutated.

The reporter plasmid for the luciferase assay, pGL4-GRE, was constructed by insertion of the GRE sequence, AGAACAgggTGTTCT, into the vector pGL4.23 (Promega, Wisconsin, WI, USA) and digested by Bgl II and Hind III. The fragment of the GRE sequences were hybridized, 5′-gatctAGAACAgggTGTTCTa-3′ to 5′-agcttAGAACAcccTGTTCTa-3′, in hybridization buffer (10 mM Tris-HCl [pH 8.0], 50 mM NaCl, 1 mM EDTA). After gel filtration by illustra™ MicroSpin G-25 Columns (GE healthcare, Little Chalfont, UK), the hybridized sequences were ligated to the vector using DNA Ligation Kit Mighty Mix (TAKARA BIO INC, Kusatsu, Japan). The plasmid encoding H2B-mCherry was kindly provided by Dr. Hiroshi Kimura. The plasmid encoding TagBFP-fibrillarin was constructed by insertion of the fibrillarin encoding sequence, which was digested by Xho I and Bam HI from the vector TagBFP-C1 (TAKARA BIO INC, Kusatsu, Japan), which was digested with the same restriction enzymes.

Laser scanning microscopy (LSM) imaging

LSM images were obtained using the LSM710-ConfoCor3 system (Carl Zeiss, Oberkochen, Germany). Before taking images for RICS, a single LSM image of EGFP-hGRs, H2B-mCherry, and TagBFP-fibrillarin was obtained (Figure 2a-d). The H2B-mCherry was used as a nuclear

marker and TagBFP-fibrillarin as a nucleolus marker. The EGFP and mCherry were excited using the 488 nm Ar^+ laser line and 594 nm HeNe laser line, respectively. The excitation light was directed to the sample by a dichroic mirror (HFT 488/594) and C-Apochromat 40x/NA 1.2 water immersion objective. The emission light was separated by a dichroic mirror (HFT 600) and separate wavelengths were detected by avalanche photodiodes (APDs) through the band-pass filter 505-530 nm for EGFP and 615-680 nm for mCherry. Sequentially, TagBFP was excited at 405 nm UV laser and its emission was detected by a META detector (415-599 nm). From this 3-color image, the region was set for RICS excluding the region of TagBFP-fibrillarin (nucleolus).

Fluorescence correlation spectroscopy (FCS)

To determine the profile of PSF, an FCS measurement was performed with a LSM710-ConfoCor3 system (Carl Zeiss, Oberkochen, Germany). Fluorescence autocorrelation function ($G(\tau)$) was acquired and fitted with the FCS Fit program by one-component models as follows:

$$G(\tau) = \frac{\langle I(t) \cdot I(t+\tau) \rangle}{\langle I(t) \rangle^2} = 1 + \frac{1 - F_{triplet} + F_{triplet} \cdot \exp(-\tau / \tau_{triplet})}{N(1 - F_{triplet})} \times \frac{1}{\left(1 + \frac{\tau}{\tau_D}\right)\sqrt{1 + \frac{\tau}{s^2 \cdot \tau_D}}} \tag{1}$$

where $F_{tripelt}$ is the average fraction of triplet state molecules, $\tau_{triplet}$ is the triplet relaxation time, F_i and τ_D is the diffusion time, N is the number of fluorescence molecules in the detection volume element defined by the lateral radius ω_0 and axial radius ω_z, and s is the structural parameter representing the ratio, $s = \omega_z / \omega_0$. From the FCS measurement of Rhodamine 6G, the lateral and axial radius was determined as 0.187 and 1.054 m, respectively. The diffusion coefficient of Rhodamine 6G was 414 $\mu m^2/s$.

Statistical analysis of diffusion coefficients

Diffusion coefficients of hGR wild type and mutants were statistically compared to each other by an f-test and a student t-test. All data shown in the histograms (Figure 3b) were used for this statistical analysis.

Coimmunoprecipitation

A day before transfection, 3×10^6 U2OS cells were seeded on 3 dishes of a 100 mm dish (Corning, Corning, NY, USA). The U2OS cells were transfected using 8 μL/dish of lipofection reagent Viafect (Promega, Wisconsin, WI, USA) and 2 μg/dish of pEGFP-hGR^{WT} and pEGFP-hGR^{D196Y}. Twenty-four hours after transfection, the 100 nM Dex (or DMSO) was added. Six hours after addition of Dex, U2OS cells were washed by cold PBS 2 times and then scraped in cold PBS and collected in 1.5 mL micro tubes by centrifugation at 6000 *g* for 5 min at 4°C. Cells were lysed by sonication in the lysis buffer CelLytic M (SIGMA-Aldrich, St. Louis, MO, USA) supplemented 1% (v/v) protease inhibitor cocktail (SIGMA-Aldrich, St. Louis, MO, USA) and 1% (v/v)

phosphatase inhibitor cocktail (Nacalai tesque, Kyoto, Japan). After centrifugation (17400 *g*, 10 min, 4°C), the supernatant was recovered. The supernatant protein concentration was determined using the Bradford Ultra reagent (Novexin Ltd, Cambridge, UK), and concentrations were adjusted by dilution. The supernatant samples were immunoprecipitated using anti-EGFP conjugated GFP-Trap_A (ChromoTec GmbH, Planegg-Martinsried, Germany) beads according to the manufacturer's instructions. Before mixing with the supernatant samples, the beads were equilibrated in cold CelLytic M. Immunoprecipitation of EGFP-GR wildtype and mutant was performed at 4°C for 6 hours by gently inverting. After removing the non-bound fraction, the beads were washed with cold wash buffer[2], 10 mM HEPES (pH 7.5), 1.5 mM $MgCl_2$, 10 mM KCl, 0.1% Nonidet P-40 , 0.5% Triton X-100 , and 150 mM NaCl. The co-immunoprecipitants were recovered by boiling in the laemmli SDS-PAGE sample buffer and then separated by SDS-PAGE using a 5-20% gradient ePAGEL gel (ATTO Corporation, Tokyo, Japan). Proteins were transferred onto an Immun-Blot PVDF membrane (Bio-Rad, Hercules, CA, USA), and membranes were blocked in PBS containing 5% (w/v) skim milk and 0.05% (v/v) Tween 20. After incubation with an anti-NCoR antibody in CanGetSignal solution 1 (TOYOBO CO., Ltd, Osaka, Japan), the membrane was incubated with anti-rabbit IgG conjugated with horseradish peroxidase in CanGetSignal solution 2 (TOYOBO CO., Ltd, Osaka, Japan). Specific binding of anti-NCoR antibodies was imaged by LAS4000mini (Fujifilm corporation, Tokyo, Japan) using the ECL Western Blotting Detection System (GE healthcare, Little Chalfont, UK) for chemiluminescence. After imaging, the antibodies were stripped from the membrane by gently shaking in the stripping buffer, 62.5 mM Tris-HCl (pH 6.7), 50 mM DTT, and 2% SDS for 2 hours at 50°C. The membrane was blocked with PBS containing 5% (w/v) skim milk and 0.05% (v/v) Tween 20. It was reblotted with anti-GR antibodies (ab3579) then imaged as same procedure as first blotting.

The band intensities were quantified with ImageJ (NIH). The tendencies of NCoR binding to the GR wild type and D196Y mutant were analyzed as relative intensity $[I_{NCoR}/I_{GR}]$ of the bands obtained from blotting with anti-NCoR and anti-GR antibodies by the following equation.

$$[I_{NCoR} / I_{GR}] = \frac{I_{NCoR} - I_{backgraound,NCoR}}{I_{GR} - I_{backgraound,GR}} \qquad (2)$$

Luciferase assay for transrepressional activity of hGR

A day before transfection, 0.7×10^6 U2OS cells were seeded on a Nunc 6-well plate (Thermo Fisher Scientific, Waltham, MA, USA). The U2OS cells were transfected using the lipofection reagent Viafect (Promega, Wisconsin, WI, USA) 3 μL/well and 1 μg/well pEGFP-hGR^{WT}, pEGFP-hGR^{C421G}, pEGFP-hGR^{A458T}, pEGFP-hGR^{I193F}, pEGFP-hGR^{L194A}, or pEGFP-hGR^{D196Y} with 0.2 μg/well pGL4-NF-κB-RE (NF-κB response element) as reporter and pNL-PGK (Promega,

Wisconsin, WI, USA) as the internal control. The pGL4-NF-κB-RE was constructed by insertion of the sequence of NF-κB-RE 3-repeat of "GGGAATTTCCGGGGACTTTCC" into the pGL4 vector (Promega, Wisconsin, WI, USA). Twenty-four hours after transfection, the 100 ng/mL LPS (Lipopolysaccharide, an activator for NF-κB) and/or 100 nM Dex (or DMSO) was added. Six hours after addition of LPS and Dex, U2OS cells were trypsinized and harvested in 1.5 mL micro tubes. After washing with cold PBS supplemented 0.8 mM AEBSF (4-(2-Aminoethyl)benzenesulfonyl fluoride hydrochloride), the luciferase assay was performed using NanoDLR Stop & Glo Luciferase Assay System (Promega, Wisconsin, WI, USA) according to the manufacturer's instructions. The chemiluminescence from the firefly and Nanoluc luciferase was measured and analyzed by Typhoon TRIO+ Variable mode imager (GE healthcare, Little Chalfont, UK) and ImageQuant TL software (GE healthcare, Little Chalfont, UK). The activity of firefly luciferase which evoked by activation of NF-κB was normalized the activity of Nanoluc luciferase using the following equation

$$Normalized\ luciferase\ activity = \frac{I_f - I_{f,background}}{I_n - I_{n,background}} \qquad (3)$$

where I_f is the intensity of firefly luciferase and I_n is the intensity of Nanoluc luciferase, $I_{f,background}$ and $I_{n,background}$ are the background intensity of measured firefly and Nanoluc luciferase activity, respectively. Then the relative transcriptional activity was calculated by using the normalized luciferase activities with LPS alone and with Dex and divided by the normalized luciferase activity with DMSO only. The mean and SD of transcriptional activities were calculated from three individual luciferase assays.

Supplementary References

1 Pack, C.; Saito, K.; Tamura, M.; Kinjo, M. Microenvironment and effect of energy depletion in the nucleus analyzed by mobility of multiple oligomeric egfps. *Biophys. J.* **2006**, *91*, 3921-3936.

2. Müller, C.B.; Loman, A.; Pacheco, V.; Koberling, F.; Willbold, D.; Richtering, W.; Enderlein, J. Precise measurement of diffusion by multi-color dual-focus fluorescence correlation spectroscopy. *EPL (Europhysics Letters)* **2008**, *83*, 46001.

3. Elbi, C.; Walker, D.A.; Romero, G.; Sullivan, W.P.; Toft, D.O.; Hager, G.L.; DeFranco, D.B. Molecular chaperones function as steroid receptor nuclear mobility factors. *Proc. Natl. Acad. Sci. USA* **2004**, *101*, 2876-2881.

4. McNally, J.G.; Muller, W.G.; Walker, D.; Wolford, R.; Hager, G.L. The glucocorticoid receptor: Rapid exchange with regulatory sites in living cells. *Science* **2000**, *287*, 1262-1265.

5. Groeneweg, F.L.; van Royen, M.E.; Fenz, S.; Keizer, V.I.; Geverts, B.; Prins, J.; de Kloet, E.R.; Houtsmuller, A.B.; Schmidt, T.S.; Schaaf, M.J. Quantitation of glucocorticoid receptor DNA-binding dynamics by single-molecule microscopy and frap. *PLoS ONE* **2014**, *9*, e90532.

6. Stavreva, D.A.; Muller, W.G.; Hager, G.L.; Smith, C.L.; McNally, J.G. Rapid glucocorticoid receptor exchange at a promoter is coupled to transcription and regulated by chaperones and proteasomes. *Mol. Cell. Biol.* **2004**, *24*, 2682-2697.

7. Schaaf, M.J.; Cidlowski, J.A. Molecular determinants of glucocorticoid receptor mobility in living cells: The importance of ligand affinity. *Mol. Cell Biol.* **2003**, *23*, 1922-1934.

8. Kino, T.; Liou, S.H.; Charmandari, E.; Chrousos, G.P. Glucocorticoid receptor mutants demonstrate increased motility inside the nucleus of living cells: Time of fluorescence recovery after photobleaching (frap) is an integrated measure of receptor function. *Mol. Med.* **2004**, *10*, 80-88.

9. Stasevich, T.J.; Mueller, F.; Michelman-Ribeiro, A.; Rosales, T.; Knutson, J.R.; McNally, J.G. Cross-validating frap and fcs to quantify the impact of photobleaching on in vivo binding estimates. *Biophys. J.* **2010**, *99*, 3093-3101.

10. Mikuni, S.; Pack, C.; Tamura, M.; Kinjo, M. Diffusion analysis of glucocorticoid receptor and antagonist effect in living cell nucleus. *Exp. Mol. Pathol.* **2007**, *82*, 163-168.

11. Mikuni, S.; Tamura, M.; Kinjo, M. Analysis of intranuclear binding process of glucocorticoid receptor using fluorescence correlation spectroscopy. *FEBS Lett.* **2007**, *581*, 389-393.

12. Kolin, D.L.; Wiseman, P.W. Advances in image correlation spectroscopy: Measuring number densities, aggregation states, and dynamics of fluorescently labeled macromolecules in cells. *Cell Biochem. Biophys.* **2007**, *49*, 141-164.

13. Wohland, T.; Shi, X.; Sankaran, J.; Stelzer, E.H. Single plane illumination fluorescence correlation spectroscopy (spim-fcs) probes inhomogeneous three-dimensional environments. *Opt. Express* **2010**, *18*, 10627-10641.

14. Robertson, S. *et al.* Abrogation of glucocorticoid receptor dimerization correlates with dissociated

glucocorticoid behavior of compound a. *J Biol Chem* **285**, 8061-8075 (2010).
